# To Disguise the Taste of Quinine

**Published:** 1886-10

**Authors:** 


### *TO DISGUISE THE TASTE OF QUININE.*

A superior method is one we would recommend. It consists of triturating equal parts of sulphate of quinine and sugar of milk, with twice the weight of crystallized phosphate of sodium, and this method can be further improved upon by the addition of saccharin, in proportion of one-fourth or one-third grain to each grain quinine, The quinine when taken in this manner is, by double decomposition, changed to a phosphate, which is soluble in only 750 parts of water.

When taken it is slightly bitter to the taste, and the phosphate of quinine is, so to say, *in statu nascenti*, and will be quickly dissolved in the gastric juice. Gelatin capsules are often objected to by children, and sometimes become insoluble. We have known them, as well as compressed quinine pills, to pass undissolved through the intestines. We know of no more elegant nor practical form of dispensing the sulphate of quinine in powder form than the inclosing in wafers. The wafers, when properly stored away and protected from insects, will keep in all climates; if carefully filled and sealed they prove superior to any modern product of *la pharmacie elegante*. After long experiments, we have, however, found the above described method of employing phosphate of sodium and the lately discovered saccharin, to be useful, and the simplest.—*National Druggist.*

